# Real-world data on the clinical impact of molecular tumor boards in high- and low-grade serous ovarian cancer

**DOI:** 10.3389/fonc.2026.1814619

**Published:** 2026-05-19

**Authors:** Fabian B. T. Kraus, Elena Benkel, Jörg Kumbrink, Kathrin Heinrich, C. Benedikt Westphalen, Celina V. Wagner, Vesela Ivanov, Andreas Jung, Martina Rudelius, Frederick Klauschen, Philipp A. Greif, Alexander König, Anca Chelariu-Raicu, Bastian Czogalla, Alexander Burges, Sven Mahner, Fabian Trillsch, Rachel Wuerstlein

**Affiliations:** 1 Department of Obstetrics and Gynecology, Comprehensive Cancer Center Munich and Bavarian Cancer Research Center (BKFZ), University Hospital, LMU Munich, Munich, Germany; 2 German Cancer Consortium (DKTK), Munich, Germany; 3 Institute of Pathology and Comprehensive Cancer Center Munich, University Hospital, LMU Munich, Munich, Germany; 4 Department of Medicine III, University Hospital, LMU Munich, Munich, Germany; 5 Comprehensive Cancer Center (CCC Munich LMU), LMU University Hospital Munich, Munich, Germany; 6 German Cancer Research Center (DKFZ), Heidelberg, Germany

**Keywords:** high-grade serous ovarian cancer, low-grade serous ovarian cancer, molecular tumor board, next-generation sequencing, ovarian cancer, precision oncology, targeted therapy

## Abstract

**Background:**

The clinical impact of molecular tumor boards (MTBs) in epithelial ovarian cancer remains insufficiently characterized, particularly with regard to rare histologic subtypes. We evaluated the real-world implementation and therapeutic relevance of MTB recommendations in high-grade (HGSOC) and low-grade serous ovarian cancer (LGSOC).

**Methods:**

We retrospectively analyzed 69 patients with HGSOC or LGSOC who underwent comprehensive molecular profiling and were presented at the interdisciplinary MTB of the University Hospital LMU Munich between November 2017 and June 2023. Molecular alterations, actionability, MTB recommendations, and clinical implementation were assessed.

**Results:**

At least one molecular alteration was detected in 50/69 patients (0.71). Actionable alterations were identified in 8/69 cases (0.12), including 4/58 HGSOC (0.07) and 4/11 LGSOC patients (0.36). MTB recommendations resulted in targeted treatment suggestions in eight patients. In three patients, treatment recommendations were clinically implemented, with a median treatment duration of 246 days (range 72–254). Non-implementation was primarily attributable to rapid clinical deterioration. LGSOC patients showed a markedly higher proportion of actionable alterations and MTB-driven therapeutic options compared with HGSOC.

**Conclusion:**

In this real-world cohort, actionable mutations in serous ovarian cancer were infrequent, particularly in HGSOC. Clinical benefit was limited by molecular targetability but also by timing and patient deterioration in late-line settings. Our findings suggest that MTB referral may be most valuable in the recurrent/refractory setting for HGSOC, whereas earlier molecular testing, potentially at first relapse, could be considered in LGSOC.

## 1 Introduction

Ovarian cancer (OC) is the most lethal among gynecologic cancers and counts as the seventh leading cause of morbidity and mortality associated with tumors in women (1, 2). Due to late symptom onset and the absence of reliable screening methods, the disease is often detected at an advanced stage, with an 85% risk of recurrence within the first decade of diagnosis (3, 4). This delay in diagnosis is associated with a significant decrease in five-year survival, from 86% in Fédération Internationale de Gynécologie et d’Obstétrique (FIGO) I patients to 26% in FIGO IV patients (5).

According to the current World Health Organization (WHO) classification, serous OC comprises two histopathologic subtypes; high-grade (HGSOC) and low-grade serous ovarian cancer (LGSOC) (6). While HGSOC is approximately nine times more prevalent and typically originates from serous tubal intraepithelial carcinoma (STIC), it is frequently characterized by *TP53* mutations (~95%) and homologous recombination deficiency (HRD; ~50%) (7). In contrast, LGSOC usually develops from borderline tumors, retains wild-type TP53, is homologous recombination-proficient, and commonly harbors Mitogen-activated protein kinase (*MAPK*) pathway mutations along with estrogen and progesterone receptor overexpression (8, 9). These biologic differences translate into divergent therapeutic vulnerabilities.

For both HGSOC and LGSOC, the cornerstone of therapy remains cytoreductive surgery followed by first-line platinum-based chemotherapy, with the extent of residual disease being the strongest prognostic factor (10–12). Since 2010, however, advances in molecular tumor profiling and the introduction of targeted therapies have reshaped the treatment paradigms for OC (13, 14) (**Table 1**).

**Table 1:** Approved and emerging molecular-targeted agents in high- and low-grade ovarian cancer.

Approved molecular-targeted agents in high- and low-grade serous ovarian cancer				
Histotype	Molecular target	Agent(s)	Setting/indication	Key references
High-grade serous Ovarian carcinoma (HGSOC)	PARP inhibition (*BRCA1/2* mutation, HRD-positive, or all-comers depending on trial/label)	Olaparib, Niraparib, Rucaparib	Maintenance therapy after response to platinum-based chemotherapy (first-line or recurrent); treatment of recurrent disease in select settings	SOLO-1 (17), SOLO-2 (18), PRIMA (19), NOVA (70), ARIEL3 (71)
	Angiogenesis (VEGF)	Bevacizumab	In combination with carboplatin/paclitaxel in front-line; as maintenance in newly diagnosed advanced disease; recurrent platinum-sensitive or -resistant disease	GOG-0218 (72), ICON7 (73), OCEANS (74), AURELIA (75)
	Folate receptor-α (FRα)	Mirvetuximab soravtansine (antibody-drug conjugate)	FDA accelerated approval (2022) for FRα-high platinum-resistant ovarian cancer after 1–3 prior regimens; confirmatory MIRASOL trial showed OS benefit	MIRASOL (76)
Low-grade Serous Ovarian carcinoma (LGSOC)	MAPK pathway (MEK inhibition; *KRAS/NRAS/BRAF* frequently altered)	Trametinib	Recurrent LGSOC (improved PFS vs physician’s choice chemotherapy/hormonal therapy; now guideline-endorsed and regulatory-approved in some regions)	GOG 281/LOGS (29), ROAR (77)
Emerging molecular targeted agents in high- and low-grade serous ovarian cancer				
High-grade serous ovarian carcinoma (HGSC)	Claudin-6 (*CLDN6*)	TORL-1-23 (ADC), zolbetuximab (mAb)	Early-phase studies in CLDN6-expressing gynecologic tumors; investigational	(78, 79)
	DNA damage response (*ATR, WEE1, PKMYT1* inhibitors)	Ceralasertib (ATRi), Adavosertib (WEE1i), RP-6306 (PKMYT1i)	Phase I-II trials; activity reported in platinum-resistant disease and PARPi-exposed patients	(80–82)
	Immune checkpoint blockade (PD-1/PD-L1, combinations)	Pembrolizumab, Dostarlimab, Nivolumab + combinations	Limited activity in unselected HGSC (KEYNOTE-100, NRG-GY003); higher efficacy in MSI-H/TMB-high rare subgroups	(26, 83)
Low-grade serous ovarian carcinoma (LGSOC)	MEK inhibition (beyond trametinib)	Selumetinib, Binimetinib	Phase II/III trials; some activity but not practice-changing; remain investigational	(84, 85)
	Combination endocrine + targeted approaches	CDK4/6 inhibitors (e.g., ribociclib, palbociclib) + letrozole	Phase II ongoing; rationale from ER positivity and cell-cycle alterations	(86, 87)
	Other MAPK-pathway inhibitors	BRAF inhibitors (dabrafenib, vemurafenib) in BRAF-mutant LGSOC		(77, 88)

In HGSOC, standard first line therapy for high-grade ovarian cancer includes six cycles of carboplatin and paclitaxel. The anti-angiogenic vascular endothelial growth factor (VEGF) inhibitor bevacizumab demonstrated improved progression-free survival (PFS) in two international phase III trials (GOG 0218 and ICON7) (15, 16) and is approved in combination with chemotherapy followed by maintenance therapy in both first-line and relapsed settings. Maintenance therapy is molecularly guided: olaparib (a Poly (ADP-ribose) polymerase (PARP) inhibitor) for *BRCA1/2* mutations, niraparib for all patients, and olaparib plus bevacizumab for HRD tumors based on the PRIMA, SOLO-1, SOLO-2, and PAOLA-1 trials (17–19). Rucaparib has also been approved as first-line maintenance therapy based on the ATHENA-MONO trial (20). In case of recurrent disease, platinum-eligible patients are re-treated with a platinum-based combination regimen ± bevacizumab or with consecutive PARP inhibitor maintenance following remission, while platinum-resistant disease is managed with non-platinum single agents (e.g., paclitaxel, pegylated liposomal doxorubicin, topotecan, or gemcitabine) ± bevacizumab, according to ASCO guidelines.

For HGSOC, emerging novel targeted therapies include antibody-drug conjugates (ADCs) (21). For instance, mirvetuximab soravtansine achieved an overall response rate (ORR) of 32.4% in a cohort of 106 heavily pretreated patients with platinum-resistant epithelial ovarian cancer and high folate receptor alpha expression (SORAYA trial) (22). Other ADCs are also being explored; for example, trastuzumab deruxtecan - an *HER2*-targeted agent - is being evaluated under a tumor-agnostic approval framework, in which eligibility depends solely on *HER2* expression rather than the tissue of origin. Consequently, its clinical activity can be assessed in *HER2*-positive ovarian cancer as part of these broader analyses (23, 24).

Although immunotherapy has had a significant impact in many cancers, anti-PD-1/PD-L1 agents remain investigational in HGSOC. PD-L1 expression and immune microenvironment profiles have shown potential correlations with response to pembrolizumab in exploratory studies, but their role as validated predictive biomarkers in epithelial ovarian cancer remains under evaluation. In the phase III ENGOT-ov65/KEYNOTE-B96 trial, the addition of pembrolizumab to weekly paclitaxel ± bevacizumab resulted in statistically significant and clinically meaningful improvements in progression-free survival irrespective of PD-L1 status and improved overall survival in patients with PD-L1 CPS ≥1 platinum-resistant recurrent ovarian cancer (25). Yet, to date, checkpoint inhibitors are still primarily recommended within clinical trials or for biomarker-selected cases (e.g., MSI-H or TMB-H), while the role of PD-L1-guided treatment selection in epithelial ovarian cancer is evolving (26, 27).

In LGSOC, the treatment options are few, as standard chemotherapies are generally less effective. This translates into increased recurrence rates and poorer long-term survival. After surgery, postoperative chemotherapy (typically a platinum-taxane combination) may be considered; particularly in advanced-stage disease, but hormonal therapy (such as aromatase inhibitors) and targeted treatments (e.g., anti-angiogenic agents like bevacizumab) play an important role, too, given the chemo-resistant nature of LGSOC. Currently, targeted therapy is increasingly shaping treatment strategies in this tumor entity. In May 2025, the FDA granted accelerated approval to the combination of avutometinib, a dual RAF-MAPK clamp inhibitor, and defactinib, a focal adhesion kinase (*FAK*) inhibitor, for adult patients with recurrent, *KRAS*-mutated disease, based on the RAMP-201 trial (28). Other biomarker-guided approaches are under investigation. The *MEK* inhibitor trametinib demonstrated a significantly improved ORR (26% vs. 6%) and prolonged PFS (13 vs. 7 months) compared with standard therapy in relapsed LGSOC (29). Given the frequent expression of estrogen and progesterone receptors, hormonal therapies have also been explored, though with limited benefit. Combinatorial regimens incorporating CDK4/6 inhibitors show promise and are currently being tested in clinical trials (30).

Despite these therapeutic advances, most biomarker-guided therapies remain investigational, and treatment decisions are frequently made without systematic molecular stratification. Molecular tumor boards (MTBs) have emerged as a central component of precision oncology, providing a structured, multidisciplinary platform to interpret complex genomic data and translate it into clinically actionable treatment strategies (31, 32). By integrating expertise from oncology, pathology, molecular biology, bioinformatics, and genetics, MTBs aim to bridge the gap between comprehensive tumor profiling and individualized patient care (33–35). This approach is particularly relevant in ovarian cancer, where late-in-line therapeutic decision-making remains challenging despite advances in targeted therapies and biomarker-driven treatments. High-grade and low-grade serous ovarian cancers represent biologically distinct entities with differing molecular landscapes, clinical behavior, and therapeutic vulnerabilities, underscoring the need for tailored treatment approaches. Although MTBs are increasingly implemented in routine practice, real-world evidence regarding their clinical impact, feasibility, and ability to influence treatment decisions in ovarian cancer remains limited (31, 36).

In this study, the real-world impact of MTB recommendations on therapeutic decision-making and clinical outcomes was assessed in patients with high- and low-grade serous OC treated at the LMU CCC University Hospital Munich.

## 2 Materials and methods

### 2.1 Precision oncology program establishment

In 2016, an interdisciplinary MTB was initiated as a cornerstone of the Precision Oncology Program at the University Hospital Munich. Its primary goal is to bring together clinical and molecular genetic patient information in comprehensive discussions that result in diagnostic and personalized treatment recommendations or clinical trial inclusion. All patients participating in our precision oncology program have consented to either of two prospective registries (The Informative Patient or SMART PRO). Both studies, conducted at LMU Munich, involved the collection of personal and clinical patient data in a central registry. Both protocols conform to the tenets of the Declaration of Helsinki and were approved by the Ethics Committee of the Medical Faculty of LMU Munich.

Starting in 2017, a comprehensive analysis of 69 cases of high- and low-grade serous ovarian cancer was conducted. All cases were first evaluated in our gynecologic tumor boards (gTB) and were subsequently referred for assessment by the MTB due to multiple prior lines of chemotherapy and increasingly limited standard treatment options. All individuals included in the study met the following criteria: (1) histologically confirmed diagnosis of HGSOC or LGSOC; (2) Eastern Cooperative Oncology Group (ECOG) performance status of 0 or 1; (3) prior assessment by the gTB resulting in recommendation for molecular tumor board presentation and (4) expressed willingness to participate in clinical trials and/or explore off-label tumor treatments.

### 2.2 Tissue harvesting and sequencing results

To ensure sample representativeness, tumor tissue was required to be obtained within two years and preferably after the last line of standard therapy, accounting for treatment-induced changes and resistance (37, 38). The median time from initiation of molecular diagnostics to completion of NGS was 17 days, and to MTB presentation 31 days.

### 2.3 Molecular genetic analysis

Molecular profiling was performed at the Institute of Pathology, LMU Munich as described in more detail in Kraus et al. (39). Tumor-rich regions were identified on H&E-stained FFPE sections and subsequent non-stained tissue sections were subjected to DNA (GeneRead DNA FFPE or QIAamp DNA FFPE advanced kits, both Qiagen) and RNA (RNeasy FFPE kit, Qiagen) extraction. Across the study timeframe, CGP was performed with multiple targeted NGS panels, reflecting the adoption of newer assay platforms (**Table 2**). These assays interrogated single nucleotide variants, small insertions/deletions, copy number alterations, RNA fusions, as well as tumor mutational burden (TMB) and microsatellite instability (MSI), where applicable.

**Table 2:** Overview of the molecular pathologic diagnostic panels employed.

Assay/panel	DNA alterations (gene number)	RNA/Gene fusions (gene number)	CNV (gene number)	TMB	MSI	Sequencing technology/provider	Cases (N)
Oncomine focus	52	23	19	-	-	IonTorrent, Thermofisher	8
Oncomine comprehensive v3	161	51	43	–	–	IonTorrent, Thermofisher	9
AmpliSeq for illumina comprehensive Panel v3	161	51	43	-	-	Illumina	20
Oncomine tumor mutation load	409	–	–	Yes	–	IonTorrent, Thermofisher	19
Oncomine comprehensive + Tumor mutation load	161	51	43	Yes	-	IonTorrent, Thermofisher; Illumina	8
Oncomine comprehensive plus	391	51		Yes	Yes	IonTorrent, Thermofisher	14
TrueSightOncology (TSO) 500	523	55	59	Yes	Yes	Illumina	8

NGS libraries were prepared using Illumina and Thermo Fisher kits according to the manufacturer’s instructions and sequenced on Ion Torrent GeneStudio S5 Prime or Illumina NextSeq 500/550 platforms. Data analysis was performed with Ion Reporter (Thermo Fisher) or Local Run Manager (Illumina). Variant annotation employed wAnnovar or VEP (variant effect predictor) and clinical variant evaluation was performed utilizing various databases including ClinVar, BRCA Exchange, gnomAD, COSMIC, and cBioPortal. Filtering was performed using a custom Python pipeline (PathInfony), and variants were manually validated in IGV. Only pathogenic, likely pathogenic, and selected VUS (allele frequency ≥3%) were reported according to HGVS nomenclature.

MTB case discussions were based on a detailed pathologic report of the NGS results, together with pathological and immunohistochemical data (e.g., programmed death ligand 1 (PD-L1), hormone receptor, and human epidermal growth factor receptor 2 (HER2)).

### 2.4 Study procedure

**Figure 1:**
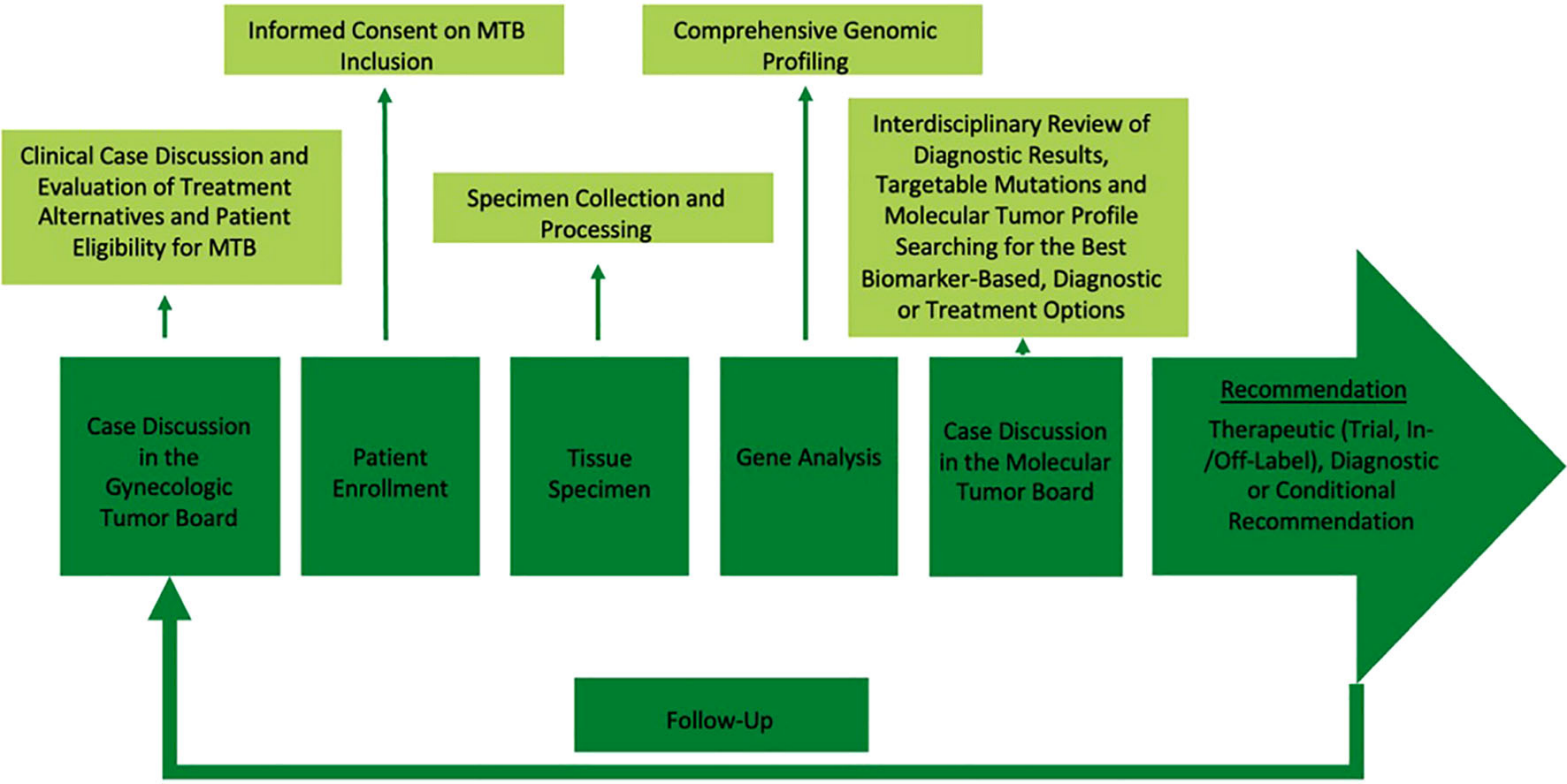
Workflow for the implementation of a Molecular Tumor Board into clinical routine.

**Figure 1** illustrates the workflow for the integration of MTB case discussions into clinical practice as recently described by Kraus et al., 2024 (39). The MTB systematically evaluated detected alterations in the presented cases to determine oncogenic relevance and therapeutic targetability. Treatment recommendations were formulated according to the ESMO Scale for Clinical Actionability of Molecular Targets (ESCAT):

Tier I (Ready for Routine Use): recommend standard-of-care targeted therapy.

Tier II (Strong Clinical Evidence, Outside Approved Indication): consider off-label therapy or clinical trial enrollment.

Tier III (Early Clinical Evidence): recommend clinical trial participation when available.

Tier IV/V (Preclinical or Limited Evidence): no immediate therapy indicated, monitor for research opportunities (40).

## 3 Results

### 3.1 Patient cohort

This study examines a cohort of 69 patients diagnosed with either high- or low-grade serous ovarian cancer, all of whom were presented to the MTB of the Department of Obstetrics and Gynecology, University Hospital, LMU Munich, Germany, between January 2018 and June 2023. The median age at initial diagnosis was 52 years (range, 24-74), and the median age at the time of MTB presentation was 57 years (range, 29-79).

At the time of MTB presentation, all patients had FIGO stage III-IV disease, the majority with metastases. The median interval from initial diagnosis to MTB presentation was 2.8 years, ranging from 0.5 to 16.0 years. Of the 69 patients included in this study, 58 (0.84) were diagnosed with HGSOC, while 11 (0.16) were diagnosed with LGSOC. All patients received a median of two lines of prior treatment regimens (range: 1 to 5) (**Table 3**).

**Table 3:** Clinical, therapeutic, and molecular characteristics of high-grade serous ovarian cancer (HGSOC, *n* = 58) and low-grade serous ovarian cancer (LGSOC, *n* = 11) cases included in the MTB cohort (*N* = 69).

	High-grade serous ovarian cancer cases (n=58)	Low-grade serous ovarian cancer cases (n=11)	Total (n=69)
Age at primary diagnosis (median, range) [a]	54.5 (24-74)	48 (33-63)	53 (24-74)
FIGO stage at primary diagnosis			
I	0	0	0
II	0	0	0
IIIA	0	0	0
IIIB	0	0	0
IIIC	10 (14.5%)	2 (2.9%)	12 (17.4%)
IVA	5 (7.2%)	0	5 (7.2%)
IVB	43 (62.3%)	9 (13.0%)	52 (75.4%)
Surgical outcome at primary surgery			
No macroscopic residual disease	36 (52.2%)	11 (15.9%)	47 (68.1%)
Macroscopic residual disease	22 (31.9%)	0	22 (31.9%)
First-line chemotherapy			
Carboplatin and paclitaxel	57 (82.6%)	11 (15.9%)	68 (98.5%)
+Bevacizumab only	37 (53.6%)	5 (7.2%)	42 (60.8%)
+Bevacizumab and PARPi	2 (2.9%)	0	2 (2.9%)
+PARPi only	3 (4.3%)	0	3 (4.3%)
Targeted medication for recurrent disease			
Bevacizumab for recurrent disease	20 (29.0%)	5 (7.2%)	25 (36.2%)
PARPi for recurrent disease	36 (52.2%)	4 (5.8%)	40 (58.0%)
Previous treatment in clinical trials	20 (29.0%)	2 (2.9%)	22 (31.9%)
Number of lines of therapy before MTB initiation (median, range)	3 (1-6)	2 (1-8)	3 (1-8)
Number of further lines of therapy following the MTB (median, range)	1 (0-5)	1 (0-4)	1 (0-5)
Molecular markers			
PD-L1 expression			
CPS = 0	5 (7.2%)	2 (2.9%)	7 (10.1%)
CPS ≥ 1	2 (3.0%)	0	2 (3.0%)
n/a	51 (73.9%)	9 (13.0%)	60 (87.0%)
*Her2* expression			
None (0)	5 (7.2%)	1 (1.4%)	6 (8.7%)
Her2 positivity (>1+)	1 (1.4%)	1 (1.4%)	2 (2.9%)
n/a	52 (75.4%)	9 (13.0%)	61 (88.4%)
Folat receptor *α* (FR *α*) expression			
negative	0	2 (2.9%)	2 (2.9%)
positive	4 (5.8%)	1 (1.4%)	5 (7.2%)
n/a	54 (78.3%)	8 (11.6%)	62 (89.9%)
Estrogen receptor expression			
negative	5 (7.2%)	0	5 (7.2%)
positive	24 (34.8%)	6 (8.7%)	30 (43.5%)
n/a	29 (42.0%)	5 (7.2%)	34 (49.3)
Progesterone receptor expression			
negative	13 (18.8%)	0	13 (18.8%)
positive	8 (11.6%)	5 (7.2%)	13 (18.8%)
n/a	37 (53.6%)	6 (8.7%)	43 (62.3%)
Mutations			
Somatic *BRCA1* status			
wildtype	52 (75.4%)	10 (14.5%)	62 (89.9%)
mutated	5 (7.2%)	1 (1.4%)	6 (8.6%)
n/a	1 (1.4%)	0	1 (1.4%)
Somatic *BRCA2* status			
wildtype	54 (78.3%)	11 (15.9%)	65 (94.2)
mutated	3 (4.3%)	0	3 (4.3%)
n/a	1 (1.4%)	0	1 (1.4%)
Mismatch repair status			
Mismatch repair deficient	0	0	0
Mismatch repair proficient	25 (36.2%)	5 (7.2%)	30 (43.4%)
n/a	33 (47.8%)	6 (8.7%)	39 (56.5%)
Tumor mutational burden (median, range)	3.8 (0-13)	3.4 (0-36)	3.8 (0-36)
*p53* status			
*p53* mutated	44 (63.8%)	1 (1.4%)	45 (65.2%)
*p53* wildtype	11 (15.9%)	10 (14.5%)	21 (30.4%)
no p53 status	3 (4.3%)	0 (0)	3 (4.3%)
*RAS* status			
*RAS* mutated	3 (4.3%)	3 (4.3%)	6 (8.6%)
*RAS* wildtype	50 (72.5%)	4 (5.8%)	54 (78.3%)
no *RAS* status	5 (7,2%)	4 (5.8%)	9 (13.0%)
Further mutations with therapeutic relevance	PIK3CA (n=1; 1.4%) PIK3R1 (n=1; 1.4%) ESR1:AKAP12 (n=1; 1.4%)	0	3 (4.3%)

Data are presented as absolute numbers, with brackets indicating either percentages or ranges as specified. Percentages are calculated based on the total cohort.

### 3.2 Molecular profiles

**Figure 2:**
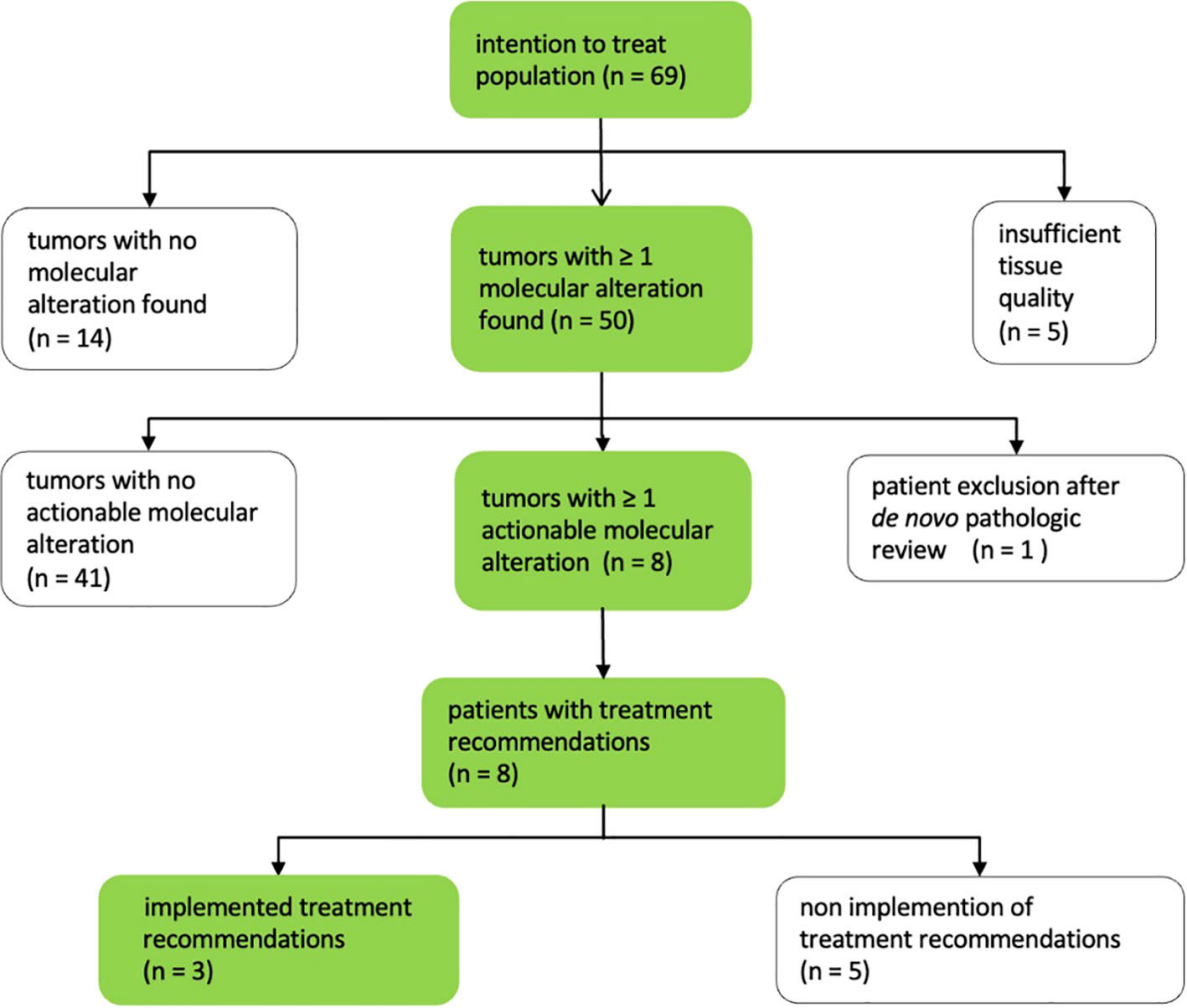
MTB output.

At least one mutation was identified in 50 of 69 patients (0.72). Of these, multiple mutations were detected in 23/69 cases (0.33). Among the 50 patients with at least one mutation, actionable molecular alterations were found in eight cases, evenly distributed between four HGSOC and four LGSOC patients. No molecular alterations were detected in the tumor specimens of 13/69 patients (0.19). Finally, genomic sequencing failed to retrieve any results in six of the 69 cases because of insufficient quality of the material (0.087) (**Figure 2**), reflecting an insufficient amount of tumor tissue for reliable sequencing.

NGS analysis revealed a total of 80 mutations in 34 different genes in the tumor samples of 64 patients with successful sequencing results, with a median of 1.25 molecular alterations per patient (range: 1 to 4). Mutations were predominantly observed in the *TP53* gene (37/80; 0.46), followed by the *KRAS* gene (4/80; 0.05), the *MYC* gene (3/80; 0.04), the *BRCA2* gene (3/80; 0.04) and the *BRCA1* gene (2/80; 0.03). A particularly high tumor mutation burden (TMB) was detected in one HGSOC patient (10 muts/Mb) and one LGSOC patient (36 muts/Mb).

### 3.3 MTB recommendations

Of the 69 patients reviewed, MTB consultations resulted in 19 recommendations for 18 individuals (0.26). These included 8/19 targeted treatment recommendations (0.42), 8/19 diagnostic suggestions (0.42), and 3/19 referrals to medical studies (0.16).

Most commonly, Ras-Raf-MAPK pathway mutations (two *KRAS* and one *Neuroblastoma RAS viral oncogene homolog* (*NRAS*) mutation) were considered targetable in three of eight patients with drug recommendations. All these patients had LGSOC and were subsequently recommended MEK inhibitors, such as binimetinib or trametinib, as biomarker-guided therapies. In the HGSOC cases, two HGSOC patients were recommended an mTOR inhibitor for a PIK3CA-AKT-mTOR pathway mutation. Due to the elevated expression of estrogen receptors in the tumor tissue of one HGSOC patient, combination therapy with everolimus and letrozole was specifically recommended. In another HGSOC case, an aromatase inhibitor was suggested by the MTB due to an *ESR1-AKAP12* fusion event.

In two additional cases, checkpoint inhibitors were recommended for a patient with HGSOC (10 muts/Mb) and a patient with LGSOC (36 muts/Mb) due to their high TMB. **Appendix A** provides a compilation of patient cases with actionable molecular alterations and the therapeutic MTB recommendations.

**Figure 3:**
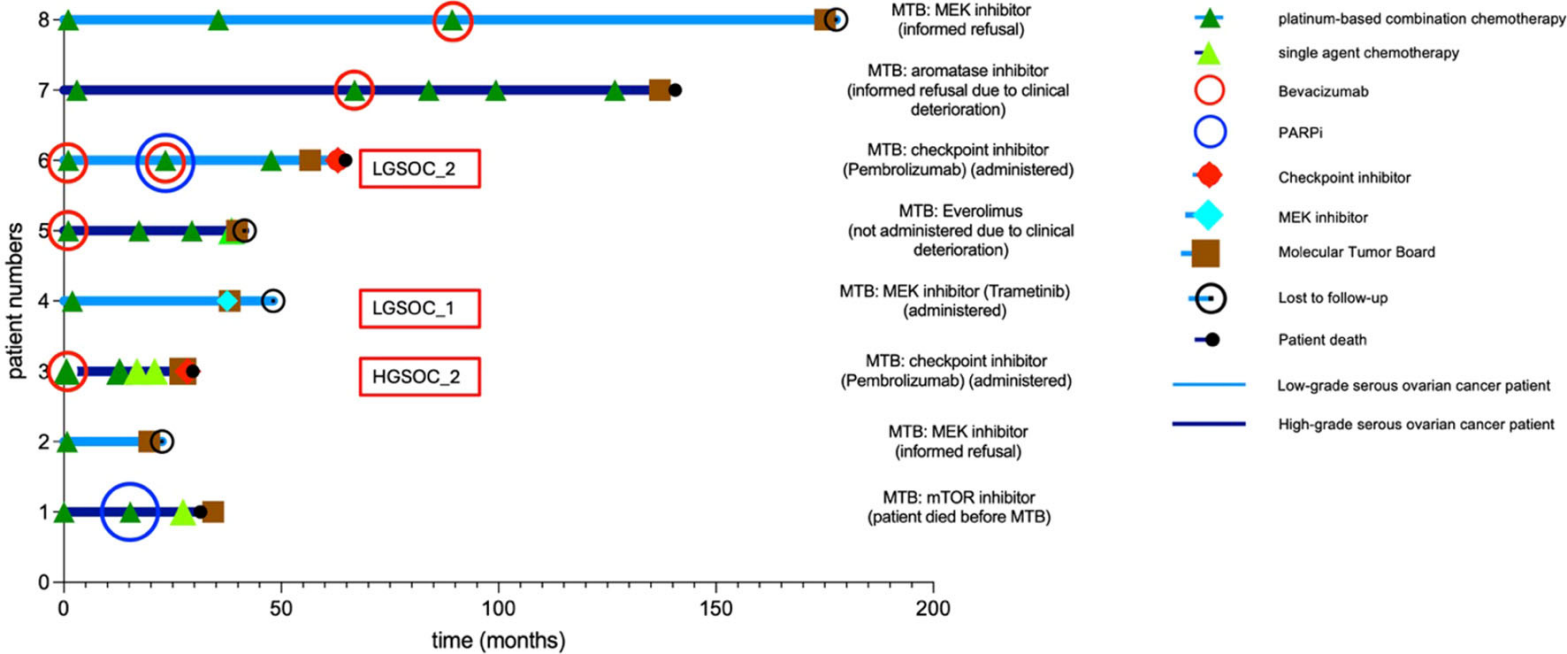
Swimmer plot of patients with therapeutic MTB recommendations, showing time in months from primary diagnosis, histological subtype (HGSOC vs. LGSOC), systemic treatments, targeted therapies (bevacizumab, PARPi, checkpoint and MEK inhibitors), MTB recommendations, and the reasons for their implementation in patient LGSOC_1, LGSOC_2 and HGSOC_1 or their non-implementation in the rest of the patients.

In total, eight out of 69 patients (0.12) received targeted drug recommendations, including four out of 58 HGSOC cases and four out of 11 LGSOC cases (**Figure 3**). Despite having an actionable *KRAS* mutation, one patient with HGSOC did not receive a targeted drug recommendation at the corresponding MTB meeting in May 2020 because Sotorasib, the first clinically approved *KRAS* antagonist, was not approved by the FDA until 2021.

In addition, three patients were recommended for enrollment in active clinical trials with inclusion criteria based on molecular information and not specifically for primary epithelial ovarian histology. One HGSOC patient was recommended for enrollment in the National Center for Tumor Diseases Study of the Safety and Efficacy of ribociclib and trametinib in Patients with Metastatic or Advanced Solid Tumors (NCT02703571). Another HGSOC patient was proposed for inclusion in the T-KNIFE clinical trial (NCT05430555). In another case of HGSOC, the MTB decided to refer the case to the Early Clinical Trial Unit (ECTU) Board of the Bavarian Center for Cancer Research (BZKF).

Out of the 69 patients, 61 (0.88) did not receive any treatment recommendation from the MTB. This was due to the following reasons: presence of non-actionable mutations in 41 cases (0.59), absence of targetable molecular alterations in 14 cases (0.20) and unsuccessful sequencing due to insufficient tissue quality in five cases (0.072) (**Figure 2**).

### 3.4 Implementation of MTB recommendations

While the gynecologic tumor board supported the MTB recommendations in all eight cases, only three MTB drug recommendations were effectively translated into clinical practice. Specifically, one patient with low-grade serous ovarian cancer (LGSOC_01) received the MEK inhibitor trametinib after complete tumor debulking as third-line adjuvant therapy, which was continued until tumor recurrence seven months later. In another case, a patient with LGSOC (LGSOC_02) underwent checkpoint inhibition with pembrolizumab as fourth-line therapy due to her high TMB until she died eight months later. Similarly, a patient with high-grade serous ovarian cancer (HGSOC_2) and high TMB was treated with pembrolizumab as fifth-line therapy until her death two months later (**Figure 3**).

In five other patients, the MTB recommendations were not implemented in clinical practice. This was primarily due to the rapid clinical deterioration observed in three patients, resulting in the immediate death of one individual and a change in treatment goal to palliative care in two others. In two further cases, the patients gave an informed refusal of the therapies suggested by the MTB (**Figure 3**).

## 4 Discussion

In recent years, advances in molecular sequencing have transformed clinical diagnostics by enabling faster and more cost-effective testing (41). Most evidence on molecularly targeted agents derives from clinical trials that pool multiple cancer types, providing broad insights but limiting disease-specific conclusions, particularly for rare malignancies. For instance, a meta-analysis of 32,149 patients with diverse cancers showed that personalized treatment was associated with higher response rates (0.31 vs. 0.10), longer PFS (5.9 vs. 2.7 months), and improved overall survival (OS, 13.7 vs. 8.9 months) compared with standard care (42, 43). However, results across studies remain inconsistent, with some analyses failing to demonstrate superiority of precision medicine (43–45). These discrepancies likely reflect tumor heterogeneity and suggest that the benefit of precision oncology is context dependent.

Recent randomized trials underscore these challenges. In CUPISCO, genomic profiling–guided therapy did not significantly improve PFS compared with standard chemotherapy in cancer of unknown primary, despite successful molecular stratification. Similarly, the ROME trial showed only modest PFS improvement with matched therapy in advanced solid tumors, with benefit largely confined to clearly actionable alterations. Together, these findings indicate that genomic matching alone is insufficient; therapeutic impact depends on tumor biology and driver strength.

The emergence of tumor-agnostic therapeutics represents a shift toward targeting molecular alterations independent of histology. Approvals for agents targeting *NTRK* fusions, MSI-H/dMMR status, *BRAF V600E*, *RET* fusions, and high tumor mutational burden exemplify this approach (46). Nevertheless, response variability across tumor types indicates that tissue-specific biology and co-mutational landscapes remain clinically relevant.

In line with this, there is a lack of cancer-specific real-world evidence on the benefits of precision medicine, particularly in rare gynecologic malignancies such as OC, and even more so in LGSOC (47–49). Therefore, our study aimed to investigate the clinical course of 69 patients diagnosed with high- or low-grade serous ovarian cancer who were evaluated at the MTB of the University Hospital of LMU Munich between January 2018 and June 2023. Of these patients, 64 (0.93) underwent successful genetic testing leading to an MTB recommendation. The size of our cohort (n = 69) is comparable to similar analyses on mixed cancer cohorts from other centers, such as the Medical University of Vienna, Austria (n = 72) (50), the Hospital Universitario Fundación Jiménez Díaz, Spain (n = 49) (51), and the University of Oklahoma Health Sciences Center, USA (n = 62) (48). Also, the clinico-pathologic characteristics of our cohort closely mirror previously reported series of serous ovarian carcinoma. As expected, LGSOC occurred at a younger age than HGSOC, with a lower median age at diagnosis in our cohort (48 vs. 54.5 years) (52). The predominance of advanced disease (17.4% FIGO IIIC, 7.2% FIGO IVA, 75.4% FIGO IVB) reflects the well-established pattern that over 70% of epithelial ovarian cancers present at FIGO stage III-IV (53). Complete macroscopic cytoreduction was achieved in 68% of patients, consistent with rates from other high-volume gynecologic oncology centers and supporting the representativeness of our surgically managed population (54). Genomic findings also aligned with published data: the prevalence of somatic *BRCA1/2* mutations in HGSOC (14%) fell at the lower end of the reported ≈ 15-30% range, likely reflecting differences in referral patterns, prior germline testing, or sequencing panels (55) *TP53* mutations were identified in 65% of successfully sequenced HGSOC samples - lower than the ≈ 80-96% in large datasets (55), but plausible in a real-world MTB setting where tissue quality, tumor cellularity, and panel coverage limit detection sensitivity. Conversely, the LGSOC profile, with RAS-pathway mutations in 27% of cases, closely matched prior reports of *KRAS*, *NRAS*, and *BRAF* alterations in 30-50% of LGSOC (52).

Building on this, in our cohort, 50 epithelial ovarian cancer patients (0.72) harbored at least one molecular alteration, irrespective of targetability. This frequency is comparable to that reported by Aust et al., who detected alterations in 86% of 44 OC cases analyzed at the Medical University of Vienna between 2015 and 2019 (47), and to findings by Spreafico et al., who identified alterations in 64% of 55 OC patients with low-grade, clear cell, or mucinous histology (7). In the Vienna cohort, targeted therapies against actionable mutations were recommended in 10% of patients (3/31; 0.10) patients (47), closely matching the proportion of actionable mutations observed in our study (8/69; 0.12).

Although these findings demonstrate the representativeness of our cohort, several limitations warrant consideration. (1) Our highly selective cohort comprised 69 patients with advanced disease who had received multiple prior therapies, limiting the availability of unexplored treatment options and the generalizability of our findings. (2) The definition of targetable mutations remains complex and requires standardized, up-to-date criteria. (3) Additionally, tumor evolution under prior therapy is critical; while we aimed to collect post-last-line tissue, ten patients declined biopsies, necessitating the use of archival material older than one year. The integration of liquid biopsy approaches could mitigate this limitation by enabling the acquisition of contemporaneous molecular data through minimally invasive sampling, likely reducing patient refusal rates and dropout while improving the timeliness and relevance of genomic profiling. Furthermore, as an observational study rather than a prospective randomized trial, these findings reflect real-world MTB-guided decision-making and should be interpreted in that context.

These challenges reflect a broader issue in precision oncology: identifying therapeutic targets and predictive biomarkers remains challenging. Even when targets are identified, clinical trials face obstacles such as slow recruitment in epithelial ovarian cancer (OC), the rarity of driver mutations, and rapid patient deterioration in late-line settings (56, 57). Molecular tumor boards (MTBs) help address these issues by stratifying patients for genotype-specific trials. Basket trials enroll patients across tumor types based on molecular alterations, while umbrella trials test multiple targeted drugs within a single cancer type (47, 50, 58). For agents such as trametinib in LGSOC, early integration into first-line therapy warrants investigation, ideally in large prospective cohorts, to improve efficacy and tolerability (59). Future trials should also define the optimal sequencing of targeted drugs, given variable responses across tumor subclones with distinct biomarker profiles.

Despite the benefits of our precision medicine program, only three of eight MTB recommendations were clinically implemented. The remaining patients either declined therapy due to rapid clinical deterioration, opted for palliative care, or died before treatment could be initiated. This highlights the challenge of determining the optimal timing for molecular testing.

Ideally, tissue samples should be obtained late in the disease course to best capture the tumor’s current genomic profile, given intratumor heterogeneity and clonal evolution under therapeutic pressure (37, 60–64). However, patients with advanced disease often deteriorate too quickly to benefit, as reported in the literature (47) and reflected in our cohort. This not only restricts access to recommended therapies (65, 66) and clinical trials (67) but also contributes to refusal of re-biopsy; for example, in a prior study, 7 of 80 patients declined biopsy, thereby precluding access to MTB (39). Furthermore, oral agents such as MEK inhibitors may be more effective earlier in treatment, as gastrointestinal absorption is often impaired in late-stage disease (30). Diagnostic delays also play a role, with a median turnaround time of 17 days (range 5-45) for next-generation sequencing (NGS), compounded by insurance approval processes. Together, these factors argue for earlier MTB presentation and streamlined diagnostics to maximize clinical benefit.

Taken together, these considerations highlight that the optimal timing of MTB referral and molecular diagnostics is critical and likely differs substantially between HGSOC and LGSOC, reflecting their distinct tumor biology, therapeutic landscapes and clinical presentation. In HGSOC, most patients initially respond to platinum-based chemotherapy combined with cytoreductive surgery, which remains the standard of care in the first-line setting (10). Biomarker testing for *BRCA1/2* mutations and homologous recombination deficiency (HRD) is now firmly established at diagnosis, as these biomarkers determine eligibility for PARP inhibitor maintenance therapy. Landmark trials such as SOLO-1 (17) and PAOLA-1 (68) demonstrated significant improvements in progression-free survival (PFS) in patients with BRCA mutations or HRD-positive tumors treated with olaparib, while SOLO-2 also reported overall survival (OS) benefits (18). Consequently, guideline-directed HRD testing is considered mandatory at diagnosis, and the incremental contribution of MTB review at this early stage is limited. Beyond these biomarkers, HGSOC is dominated by *TP53* alterations, copy-number variations, and complex genomic rearrangements, which currently lack direct therapeutic implications in most cases (7). MTB referral in HGSOC is therefore most relevant in the recurrent or treatment-refractory setting, once standard options have been exhausted. In this context, MTBs provide access to clinical trials, basket or umbrella studies, and biomarker-driven off-label treatments. They are particularly valuable for rare but actionable events, such as *Neurotrophic Tyrosine Receptor Kinase* fusions, *HER2* amplifications, high TMB, or MSI, which may render patients eligible for tissue-agnostic therapies, including immune checkpoint inhibitors.

In contrast, the role of MTBs is more promising in LGSOC, which in our dataset is reflected by a 50% rate of MTB recommendations for LGSOC patients as opposed to a rate of 7% in HGSOC. Unlike HGSOC, LGSOC is genomically stable, *TP53* wild-type, and frequently harbors activating mutations in the MAPK signaling pathway (e.g., *KRAS*, *NRAS*, *B-Raf proto-oncogene*) (69). Importantly, these alterations are therapeutically targetable. The phase III GOG-281/LOGS trial demonstrated that the MEK inhibitor trametinib significantly improved PFS (13.0 vs. 7.2 months) and overall response rate (26% vs. 6%) compared with physician’s choice chemotherapy or hormonal therapy in relapsed LGSOC (29). Given the poor chemotherapy responsiveness of LGSOC and the younger median age of affected patients, early MTB integration is crucial to ensure timely molecular testing and access to MAPK pathway-directed therapies. Notably, the combination of avutometinib (VS-6766) and defactinib has received accelerated approval by the U.S. Food and Drug Administration (FDA) for the treatment of adult patients with *KRAS*-mutated recurrent LGSOC who have received prior systemic therapy (28). This approval underscores the importance of MTBs in facilitating adaptive treatment planning and supporting patient enrollment in clinical trials.

## Data Availability

The raw data supporting the conclusions of this article will be made available by the authors, without undue reservation.
